# A Bibliometric Analysis of the Landscape of Pediatric Liver Transplantation

**DOI:** 10.3389/fped.2022.805216

**Published:** 2022-04-11

**Authors:** Lei Shi, Jie Zhou, Chenyi Jiang, Wanbing Dai, Weifeng Yu, Qiang Xia, Diansan Su

**Affiliations:** ^1^Department of Anesthesiology, School of Medicine, Renji Hospital, Shanghai Jiao Tong University, Shanghai, China; ^2^School of Medicine, Shanghai Jiao Tong University, Shanghai, China; ^3^Department of Liver Surgery, School of Medicine, Renji Hospital, Shanghai Jiao Tong University, Shanghai, China

**Keywords:** pediatric liver transplantation, bibliometric analysis, hotspots, research front, web of science

## Abstract

**Background:**

Nowadays, pediatric liver transplantation (PLT) has become an effective strategy for treating various acute or chronic end-stage liver diseases and inherited metabolic diseases. Many experts have already concluded the current challenges and future directions of PLT. However, no detailed analysis of the publication landscape has substantiated these proposed opinions.

**Methods:**

This study presents a bibliometric review of the articles related to PLT between 1997 and 2020. A total of 3,084 publications were analyzed mainly by CiteSpace and VOSviewer.

**Results:**

The field of PLT has evolved gradually in the past two decades. Articles increased at an average rate of 97 articles every 4 years. University of Pittsburgh (PITT) is the most prolific institution. The three most productive regions are North America, Europe, and East Asia. Currently, interdisciplinary studies on PLT are scarce. The main goal of PLT has shifted from survival rates to long-term outcome. The quality of life, living donor liver transplantation (LDLT), immunological biomarkers, perioperative hemodynamic management, expanding the indications of PLT, etc. are parts of the emerging research fronts. In the past two decades, articles that contain potentials that may lead to transformative discoveries are scarce, and obvious deficits can be seen in the field of new therapies.

**Conclusions:**

Long-term outcome and good quality of life represent the principal direction of work concerning PLT. Deficits in new therapies align with the shortage of intellectual milestones, which indicate possible subsequent intellectual milestones may occur as innovations in therapies such as new immunosuppression therapies or liver cell transplantation.

## Introduction

Since the first successful pediatric liver transplantation (PLT) was operated by Thomas Starzl in 1967, patient survival rate has increased markedly by the introduction of immunosuppression therapy and improvement of surgical techniques. Nowadays, PLT has become an effective strategy for treating various acute or chronic end-stage liver diseases and inherited metabolic diseases (1).

However, the success in PLT is also accompanied by additional challenges. For example, because of the long-term patient survival and the development of test methods, more long-term problems have been discovered, such as the increased prevalence of medication non-adherence (2) and hepatitis-related fibrosis (3). Due to its effectiveness in treating various liver diseases, more conditions are requiring PLT, such as α1-Antitrypsin deficiency (4) and non-alcoholic fatty liver disease (5). This leads to increased demands, which will cause a shortage of donor organs, asymmetric competition for organs between children and adults, and modification of the current allocation algorithm (6).

Considering how important PLT is to children with liver diseases, many experts have already made efforts to review the current state of and new advances in PLT (7, 8). Expert opinions are crucial, and in most cases, are coincident. However, when it comes to controversial issues or areas that have not yet aroused extensive discussion, we may need additional information to help us come to our conclusions and make decisions. Thus, a detailed bibliometric analysis of the existing research publication landscape is necessary. Bibliometrics is a statistical method that can analyze a large number of publications. By using appropriate analytic means and indicators that can underscore potentially significant content, it can provide an overview of the trends in PLT publication and insights on where the evidence is robust and where opportunities and study deficits exist (9).

This report presents a bibliometric review of the articles on PLT based on the data obtained from the Web of Science Core Collection. It seeks to identify the publication trends, shifts of hotspots and research fronts, and intellectual milestones in the field of PLT. Furthermore, it also seeks to explore how these shifts happened and what kind of researches can be new milestones. The results of this work may be helpful in future research planning and decision-making.

## Materials and Methods

Relevant publications were extracted from the Web of Science Core Collection utilizing the following search strategy:

TOPIC: ((((child^*^ near/3 (liver near/2 transplant^*^)) or (child^*^ near/3 (hepat^*^ near/2 transplant^*^))) or ((p$ediatric^*^ near/3 (liver near/2 transplant^*^)) or (p$ediatric^*^ near/3 (hepat^*^ near/2 transplant^*^)))))

The use of “^*^” means any group of characters. The use of “$” represents 0 or 1 character. Using “NEAR/x” means finding records where the terms joined by the operator are within a specified number of words of each other.

Refined by: DOCUMENT TYPES: (ARTICLE)

Timespan: 1997–2020.

The main reason for using this strategy is that we want to include as many articles related to PLT as possible. To minimize bias, literature retrieval and data downloads were completed within 24 h on June 7, 2021. The full records of the search results were downloaded in TEXT format. CiteSpace 5.8 and VOSviewer 1.6.16 were the main tools used to analyze the data. CiteSpace, the abbreviation of “Citation Space,” is a software developed by Dr Chaomei Chen for visual analytic tasks of science mapping, including collaboration network analysis, co-word analysis, document co-citation analysis, etc (10). It is one of the few software tools that can construct document co-citation networks and is widely used in scientometric studies. VOSviewer is also a bibliometric software tool used in map creation from network data, and it is useful in map visualization and exploration.

First, an overview of the publications in the field of PLT was made. Networks of institutions and regions that can depict the collaboration status of PLT were constructed. Furthermore, to learn multidisciplinary cooperation in PLT, dual-map overlay, which reveals arrangements of a scientific collection in relation to a global map of scientific literature, was used.

By detecting hotspots, recent and frequently discussed topics and their shifts were explored. Burst detection, a computational procedure useful in identifying sudden changes of events and other types of information (11), was done since the burstiness of keywords is an important indicator of most active research topics (12). Meanwhile, the term co-occurrence network was created to support the findings in burst detection and further elucidate the structure of the terms.

In many cases, research fronts are consistent with the hotspots. However, some emerging areas may not arouse scholarly interest as soon as they appear; hence, detecting hotspots alone may overlook them. Thus, analyzing the research fronts cannot be skipped. Based on the theory that the pattern of bibliographic references indicates the nature of the scientific research front (13), major areas of research activities concerning PLT were presented as clusters in the co-citation network. Considering that excluding ongoing studies may cause important information to be missed, the most recent PLT clinical trials on ClinicalTrials.gov were searched to extract their main points. The study status was limited to “recruiting, not yet recruiting, or active not recruiting” with a start date below January 1, 2018. The search date was June 19, 2021.

Intellectual milestones are the most important articles in one field. Extracting their main points reveals a deeper understanding of the development of PLT. In the co-citation network, cited articles that were identified by structural and temporal metrics of research impact and evolutionary significance were noted. Cited articles with the strongest citation bursts were chosen to analyze their main points. For more recently cited articles, Plum Analytics was used. Plum Analytics is an altmetrics tool that provides a measure of peoples' interaction with certain research outputs by collecting PlumX Metrics.

The dual-map overlay, burst detection and co-citation network were created using CiteSpace. Networks of institutions and regions and the term co-occurrence network were created using VOSviewer.

## Results

### An Overview of PLT Publications

**Figure 1:**
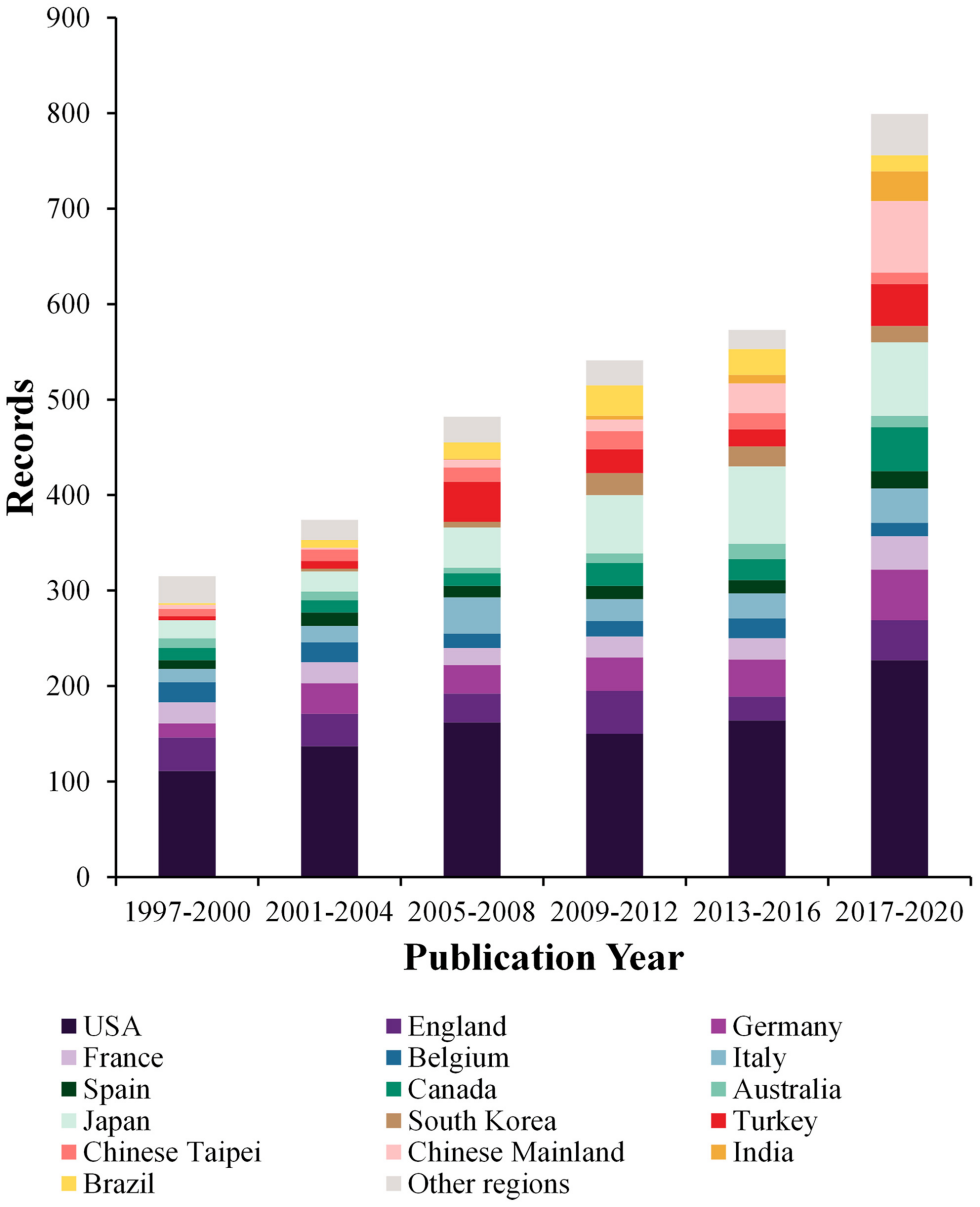
Quantitative growth process of the studies from different countries and regions concerning pediatric liver transplantation (PLT) in 24 years.

**Figure 2:**
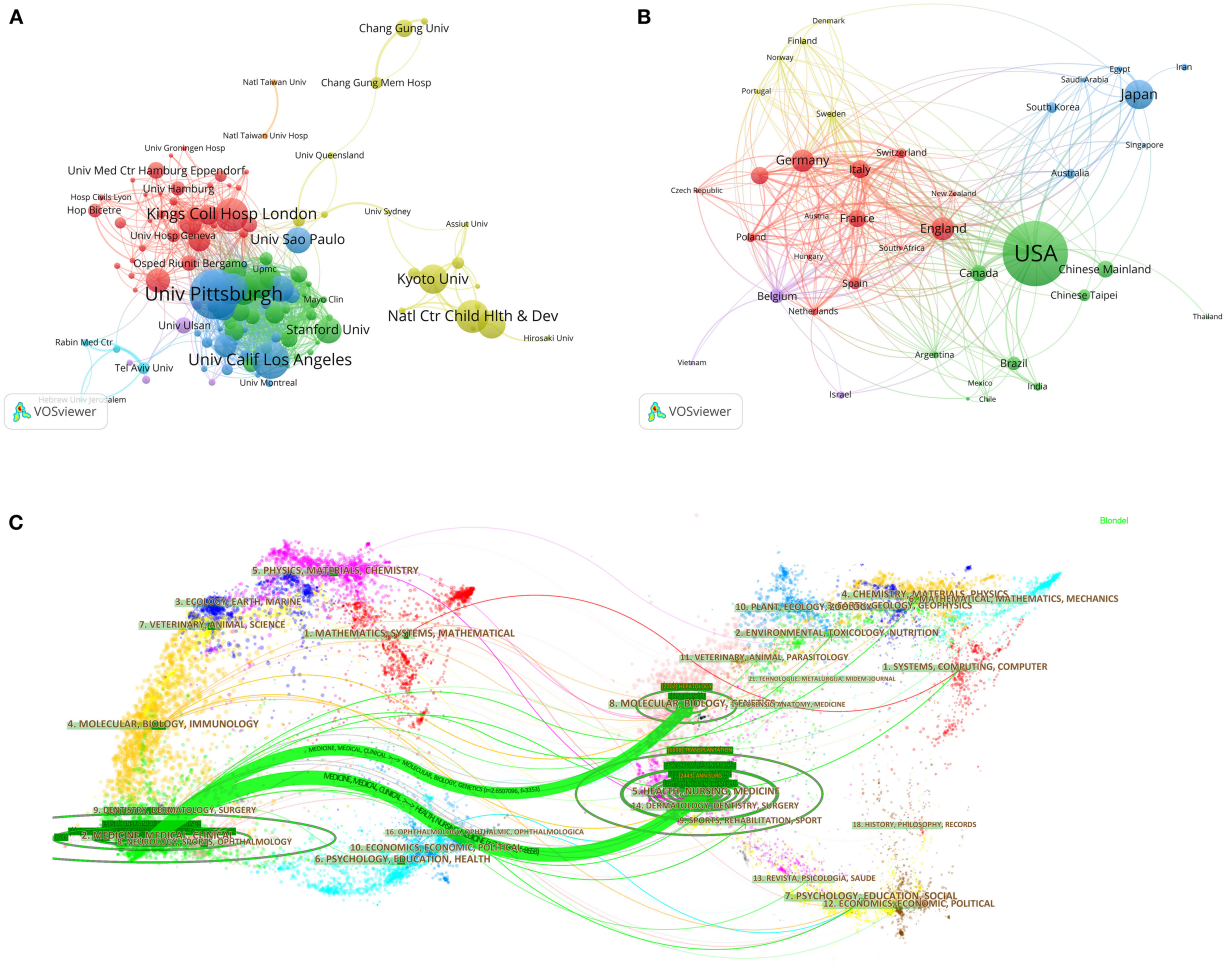
The collaboration status of pediatric liver transplantation (PLT). **(A)** Network of institutions created by VOSviewer. The resolution in the action panel was set at 0.8. The weight in the options panel was set as “documents.” Node size means the occurrence times of the institutions. **(B)** Network of countries/regions created by VOSviewer. The resolution in the action panel is set at 0.8. The weight in the options panel is set as “documents.” Node size means the occurrence times of the regions. **(C)** Dual-map overlay of the literature about PLT. Each dot on the left or right side represents a journal. The dots on the left side compose the citing journal map, and the dots on the right side compose the cited journal map. Labels are extracted from journal titles and show the disciplines involved in the series. The lines are citation links, beginning from the left and pointing to the journals on the right. The size of the oval means how many people are in terms of authors and how many papers are published in those areas.

After the removal of duplicate articles, 3,084 results were included from the Web of Science Core Collection published between 1997 and 2020. As shown in Figure 1, the findings indicate that over the past 24 years, there has been a relative growth of studies on PLT, such that the number of records rose from 315 to 799, increasing at an average rate of 97 articles every 4 years. In addition, we can find that the proportion of studies in developing countries such as China and India are gradually increasing. Primary institutions and their collaborations in the field of PLT are shown in Figure 2A. University of Pittsburgh (PITT) is the most prolific institution in the field of PLT, contributing 102 documents and 3,398 total citations. The University of California, Los Angeles is ranked second in terms of publication number. In the network of regions (Figure 2B), the three most productive regions in terms of research outputs in the field of PLT are North America, Europe, and East Asia. Besides, the US is the most productive country with 965 documents. Japan is in the second position.

The dual-map overlays are designed by Chen and Leydesdorff to reveal patterns of a scientific portfolio with respect to a global map of scientific literature (10). The dots on the left side compose the citing journal map and the right sided dots compose the cited journal map. Labels are extracted from journal titles and show the relevant disciplines involved in the series. The lines are citation links, beginning from the left and pointing to the journals on the right. The dual-map overlay presents that almost all the articles about PLT were published in one discipline (“medicine medical clinical”). Publications in the field of PLT are mainly built on two disciplines on the right side of the map (“health nursing medicine” “molecular biology genetics”) (Figure 2C).

### Hotspots in PLT

**Figure 3:**
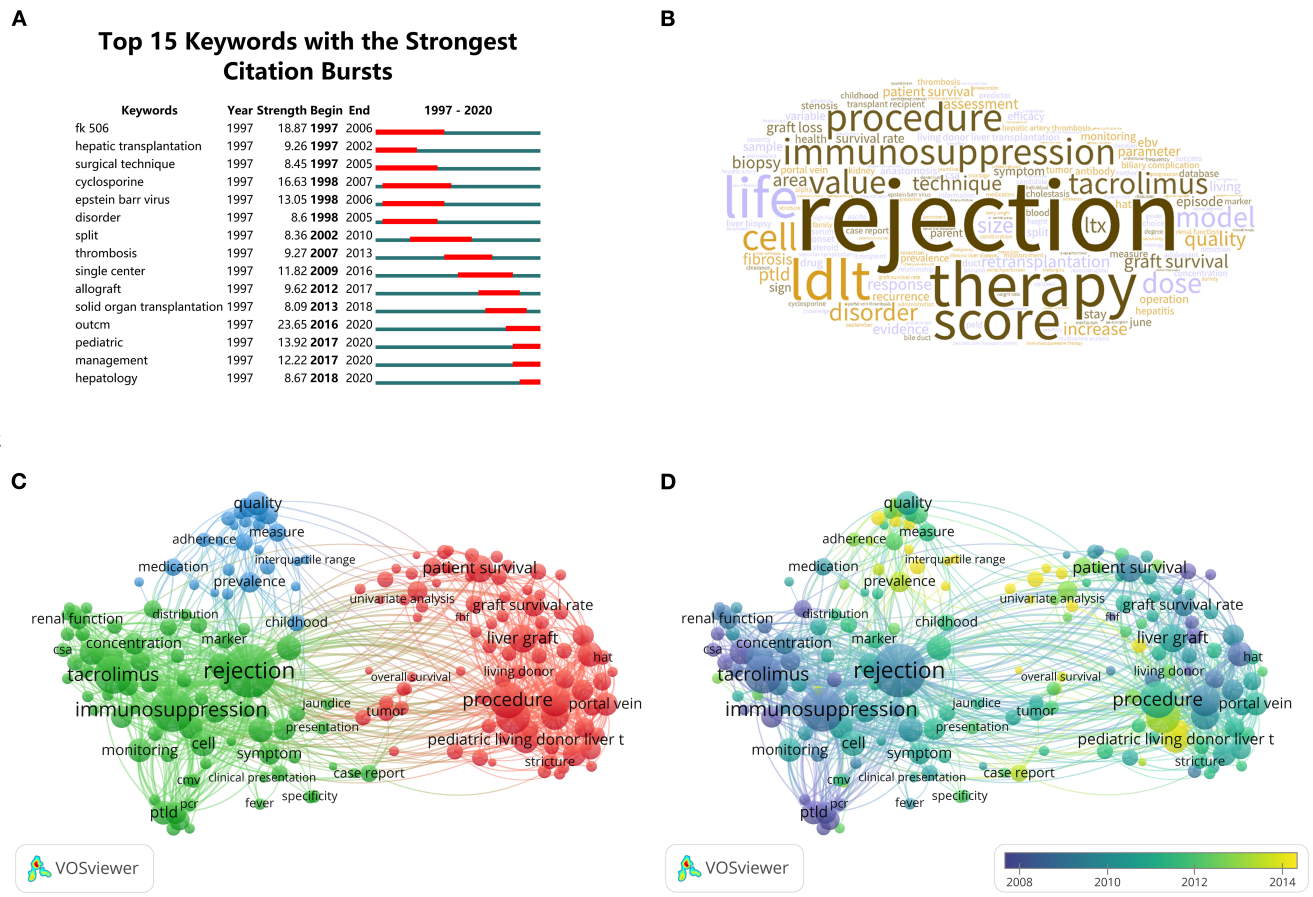
Hotspots in pediatric liver transplantation (PLT). **(A)** Top 15 keywords with the strongest citation bursts. **(B)** Word cloud of terms of PLT. The sizes of the words are proportional to their occurrence times. **(C,D)** Term co-occurrence network created by VOSviewer. **(C)** is the network visualization, and **(D)** is the overlay visualization. The resolution in the action panel is set at 0.85. The weight in the options panel is set as “total link strength.” Circle size means the occurrence times of the terms. In **(C)**, circles are colored according to their clusters. In **(D)**, circles are colored differently according to their average occurrence time.

Hotspots can be regarded as one or several topics that scholars in certain disciplines commonly pay attention to. In CiteSpace, burst detection showed keywords that increased suddenly over time so that the burstiness of keywords is an important gauge of most active research topics (12). The time interval is represented as a blue line. The period time in which a keyword was detected to have a burst is revealed by the red line segment, demonstrating the year span of the burst. In Figure 3A, keywords with maximal burst strength before 2010 are tacrolimus (FK 506) and cyclosporine A (CsA), and after 2010, the keywords are outcome, pediatric, and management. This may suggest the transition of hotspots from immunosuppression to prognosis.

After extracting terms from the title and abstract field by VOSviewer, a brief view of the most mentioned terms in the past 24 years by a word cloud was observed (Figure 3B). The biggest word in the center of the word cloud is rejection, suggesting that minimizing rejection and induction of tolerance may be a crucial issue for a long time. Constructing the term co-occurrence network by VOSviewer revealed information such as the connection between the terms. As shown in Figure 3C, terms can be roughly grouped into three clusters namely: clusters concerning immunosuppression therapy and its complications (green), clusters regarding surgical procedure and its complications (red), and clusters involving long-term outcomes (blue). In Figure 3D, the circles are colored differently according to their average occurrence time. Comparing Figures 3C,D, it is easy to find that the average occurrence time of terms concerning long-term outcomes is much later than those concerning the other two clusters, which is consistent with the results from burst detection.

### Research Fronts in PLT

**Figure 4:**
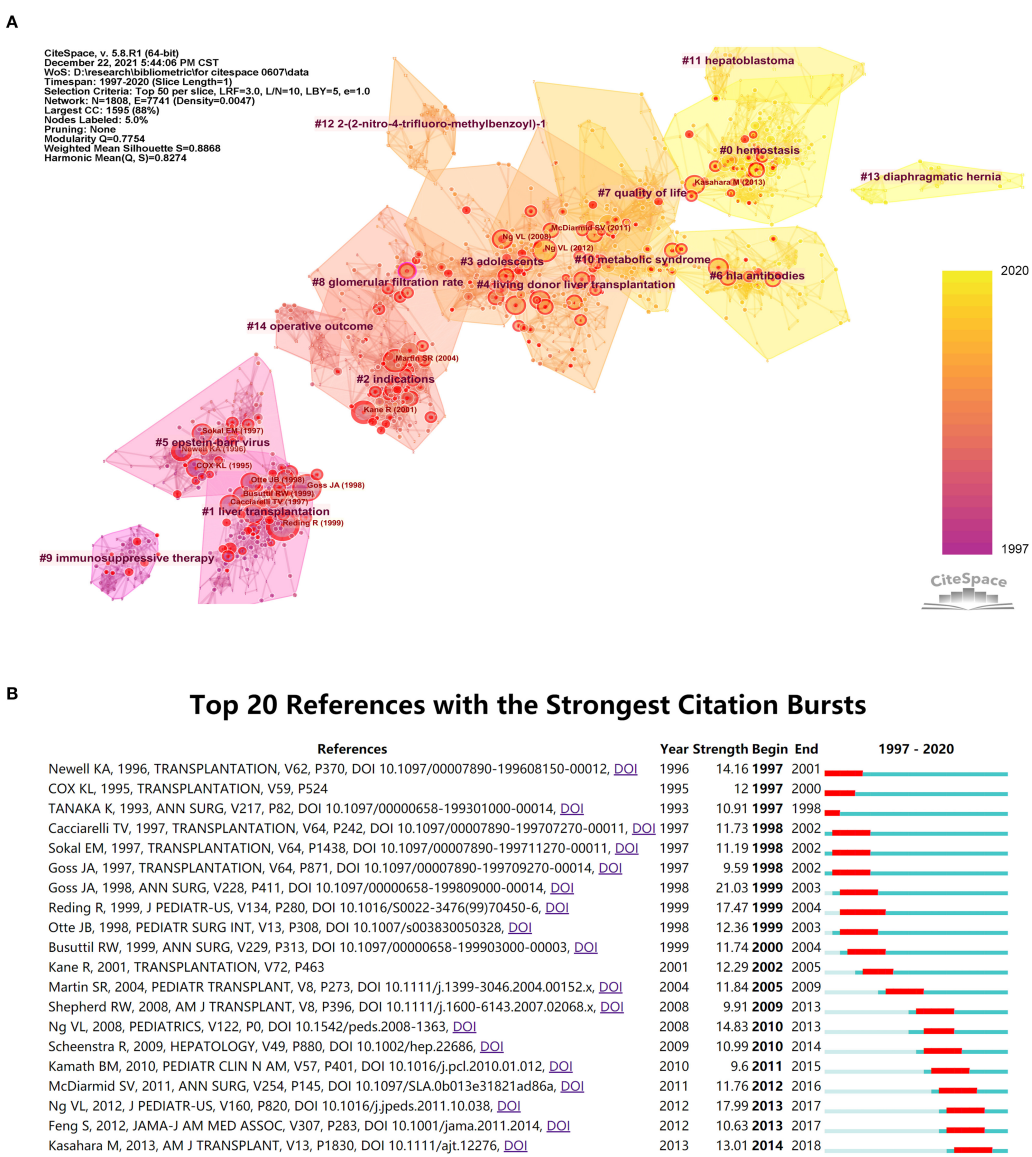
Research fronts in pediatric liver transplantation (PLT). **(A)** A landscape view of the co-citation network, generated by top 50 per slice between 1997 and 2020. The network has modularity of 0.77, implying that the specialties are clearly defined. The average silhouette score is 0.88, suggesting that the homogeneity of the network is high. The nodes in the network are cited articles, and the links are the co-citation relationship between cited articles. Node sizes are proportional to the cited times of the articles. The areas of varied colors indicate the time when co-citation links in those areas appeared for the first time. **(B)** Top 20 references with the strongest citation bursts.

Research fronts are the emerging theories or research subjects. According to the theory of Derek John de Solla Price, the pattern of bibliographic references indicates the nature of the scientific research front (13). Therefore, the constructed co-citation network revealed the evolution of the major specialties in PLT and the current research fronts. Figure 4A is the landscape view of the co-citation network. The nodes in the network are cited articles, and the links mean the co-citation relationship between cited articles. Node size indicates the number of times the article was cited. The various colors specify the time when co-citation links in those areas initially appeared. For example, purple areas were generated before the orange parts. Analyzing the label and color of clusters together with the citing and cited articles in each cluster, we can find that the research theme of PLT gradually changed from 1997 to 2020. During the first half of the period, most of the researches focused on themes about immunosuppressive therapy, operative therapy, and their short-term complications; whereas during the second half of the period, the rising specialties in PLT included quality of life, living donor liver transplantation (LDLT), immunological biomarkers, perioperative hemodynamic management, and expanding the indications of PLT, such as PLT for metabolic liver diseases (MLDs) and hepatoblastoma.

However, using the co-citation network to detect the scientific research front may cause the non-inclusion of certain relevant ongoing studies. Therefore, ongoing studies about PLT were searched on ClinicalTrials.gov. After reading the descriptions of all 31 results and excluding one irrelevant research, the themes of the remaining 30 studies were categorized (Table 1). Three of the top four themes were consistent with the results in CiteSpace, namely risk factors, observation of the long-term outcome, and improving adherence. Besides, in theme 1, three of five studies were found concerning COVID-19 in PLT recipients, which were related to the outbreak of COVID-19 since 2019. Some research themes, such as liver cell transplantation, were undetected in CiteSpace. These themes may also represent promising directions.

**Table 1:** Clinical trials categorized by their research theme.

**Theme number**	**Research theme**	**Count**
1	Immunosuppression-related virus infection	5
2	Risk factors	5
3	Observation of the long-term outcome	4
4	Improving adherence	4
5	Improving perioperative management	4
6	Improving intraoperative management	3
7	Liver cell transplantation	1
8	New imaging method	1
9	Metabolic liver diseases	1
10	Implementation of PLT^*^ biobank	1
11	Preventing specific complication	1

* *PLT, pediatric liver transplantation*.

### Intellectual Milestones in PLT

Intellectual milestones in one field can not only help growing readers better understand the field but also help the professional researchers learn more about studies that have a better impact. In CiteSpace, important articles are identified as those with high betweenness centrality or citation burstiness. High betweenness centrality indicates the structural importance of a cited article to the co-citation network. Cited articles having high betweenness centrality usually appear in the network as nodes which link two or more clusters. Cited articles having high betweenness centrality, which are also regarded as the “turning point” in CiteSpace, connect different research fields, so that they have the potential to create a new research field. Cited articles with high betweenness centrality will be reflected in the network by coloring the nodes corresponding tree ring by purple. For cited articles that exhibit citation bursts, the corresponding tree ring will be colored in red. Although in Figure 4A, many articles demonstrated citation bursts, few of them had high betweenness centrality, which suggests that the PLT field may currently lack studies containing potentials that may lead to transformative discoveries.

Since cited articles with strong citation bursts mean that they hit what the PLT field was interested in at that time, we then did the burst detection and chose the top 20 cited articles with the strongest citation bursts (Figure 4B). Interestingly, after comparing Figures 4A,B, we found that these articles were almost gathered in two periods, around 1997 and 2012. For those around 1997, we chose four with the strongest citation bursts to analyze (Table 2). Then, for those around 2012, six articles with the strongest citation bursts were chosen to be analyzed. Because these articles are closer to the present, we used altmetrics for the analysis. Over recent decades, social media has developed into a fundamental part of scholarly communication, mainly *via* the academic use of social media platforms (18). Therefore, altmetrics which is set on these activities could provide wider and quicker measures of impact, augmenting traditional citation metrics (19). The PlumX Metrics of the six cited articles were collected on July 12, 2021 (Table 3). Undoubtedly, the study conducted by Feng (25) is the most influential study, with high scores in almost every category, especially in citation and social media, which means both professionals and non-professionals are interested in this study.

**Table 2:** Four articles with the strongest citation bursts around 1997.

**Number**	**Title**	**Burst**	**Year**
1	Long-term results of pediatric liver transplantation: an analysis of 569 transplants (14)	21.03	1998
2	Pediatric liver transplantation with cadaveric or living related donors: comparative results in 90 elective recipients of primary grafts (15)	17.47	1999
3	Posttransplant lymphoproliferative disease in pediatric liver transplantation. Interplay between primary Epstein-Barr virus infection and immunosuppression (16)	14.16	1996
4	Pediatric liver transplantation: from the full-size liver graft to reduced, split, and living related liver transplantation (17)	12.36	1998

**Table 3:** Six articles with the strongest citation bursts around 2012.

**Number**	**Title**	**Burst**	**Year**	**PlumX metrics**
				**Citations^A^ / Usage^B^ / Captures^C^ / Mentions^D^ / Social media^E^**
5	Health status of children alive 10 years after pediatric liver transplantation performed in the us and canada: report of the studies of pediatric liver transplantation experience (20)	17.99	2012	147 / 236 / 145 / 0 / 0
6	Outcomes of 5-year survivors of pediatric liver transplantation: report on 461 children from a North American multicenter registry (21)	14.83	2008	167 / 962 / 146 / 0 / 0
7	Long-term outcomes of pediatric living donor liver transplantation in japan: an analysis of more than 2,200 cases listed in the registry of the japanese liver transplantation society (22)	13.01	2013	92/ 123/ 67/ 0/ 1
8	A multivariate analysis of pre-, peri-, and post-transplant factors affecting outcome after pediatric liver transplantation (23)	11.76	2011	81 / 32 / 55 / 0 / 0
9	Graft fibrosis after pediatric liver transplantation: 10 years of follow-up (24)	10.99	2009	123 / 17 / 38 / 0 / 0
10	Complete immunosuppression withdrawal and subsequent allograft function among pediatric recipients of parental living donor liver transplants (25)	10.63	2012	233 / 709 / 112 / 0/ 21

A *“Citations” is a category that contains both traditional citation indexes and citations that help indicate societal impact*.

B *“Usage” is a way to signal if anyone is reading the articles or otherwise using the research*.

C *“Captures” indicates that someone wants to come back to the work*.

D *“Mentions” is the measurement of activities such as news articles or blog posts about research*.

E *“Social media” includes the tweets, Facebook likes, etc. that reference the research*.

## Discussion

The purpose of this article was to provide a detailed bibliometric analysis of global research on PLT. Given how important PLT is to children with liver diseases, it is necessary to create an overview of the status of publications and a clear picture of scientific exchanges in the field because of its significance in helping research planning and decision-making.

Over the past two decades, there has been a prominent growth of studies on PLT, indicating that this field is evolving steadily. A pronounced increase in publications is noted after 2017 (Figure 1). This increase may be attributable to the abrupt increase of articles from developing countries, especially the Chinese Mainland. The accumulation of experience (26, 27), the promotion of donation after cardiac death (28), and the rapid growth of LDLT (29) in the Chinese Mainland in the past decade may be the accompanying reasons. University of Pittsburgh (PITT) is the most prolific institution in the field of PLT. This is because PITT has a long PLT history and has many pioneers in the field of PLT, such as Thomas Starzl. The three most productive research regions are North America, Europe, and East Asia. The US is still the most productive and influential country now. Countries/regions in East Asia seem to have fewer international collaborations than those in North America and Europe. The intellectual base of most studies is still from disciplines concerning biomedicine, but many experts are devoted to and appealing for interdisciplinary studies, such as liver transplantation combined with materials science (30), computer science (31, 32), sociology, and ethics (6, 33). This possibly hints that because of the lack of interdisciplinary studies, attempts to combine PLT with multiple disciplines may be promising.

Through analyzing the hotspots and research fronts, we can have a rough understanding that the evolution of the PLT researches involves the development of many interrelated specialties. At the early stage, people were mainly devoted to improving immunosuppression therapy and surgical procedure. This is due to the unsatisfactory survival rate after PLT at that time. Before 1980, between 50 and 71.2% of all recipients of liver transplants died during the first postoperative year (34). This discouraging status was improved greatly in the 1980s by the introduction of CsA, a new immunosuppressive agent that almost doubled the 1-year survival rate, whereas the acquisition of more surgical experience played a relatively less significant role in improving the survival rate (35). In the 1990s, tacrolimus, a more potent immunosuppressive agent, was widely introduced in PLT, which explained why many studies concerning the comparison between tacrolimus and CsA sprang up in the 1990 and 2000s (Figure 3A). However, all subsequent refinements in immunosuppression therapy failed to recreate a great success such as that of the introduction of CsA. Subsequently, the survival rate grew steadily, and the 1-year survival rate reached over 90% in the 2010s (36). Therefore, the main goal of PLT shifted to good quality of life, and studies about it will be the principal direction in PLT in the 2010s and 2020s. These studies include observation of long-term outcomes, identifying risk factors especially immunological biomarkers, improving adherence (Figure 4A), etc. Meanwhile, since PLT has become an effective strategy for treating various liver diseases, another group of studies is emerging due to the ever-increasing PLT needs, which include LDLT and expanding the indications of PLT for MLDs and hepatoblastoma (Figure 4A). Among these research fronts, obvious study deficits can be found in the area of new therapy, which may be most effective in solving the current crisis in PLT. For example, some evidence has shown that liver cell transplantation may be an alternative to organ transplantation for acute liver failure and liver-based metabolic deficiencies (37–40), but studies on it are still relatively scarce. Thus, it is rational to infer that studies of new therapies like new immunosuppression therapy or liver cell transplantation will become future research fronts.

Early intellectual milestones mainly revolved around refinements in immunosuppression and surgical techniques. The studies of Reding (15), Newell (16), and Otte (17), respectively answered questions about the choice of liver source, immunosuppression strategy, and surgical procedure. Unlike other early intellectual milestones, Goss (14) conducted one of the earliest studies about long-term results. It is a single-center study and its definition of long-term outcome is limited to the patient survival rate and complications, but in the 1990s, it just answered the question about the PLT long-term outcomes. We regarded these questions as what researchers in the PLT field most cared about before 2000. For intellectual milestones around 2012, Feng' study is one of the earliest multi-center studies to assess the feasibility of immunosuppression withdrawal (25). Immunosuppression withdrawal aroused great interest around 2012, probably as a result of the increased prevalence of medication non-adherence (2) and the desire to avoid immunosuppression-related adverse reactions (41, 42). In CiteSpace, no significant “turning points” were found. This may be related to the slicing method that we used, which decides how to divide the time span of research and how many articles should be included in each slice, but a more reasonable explanation is that after the pioneering years when immunosuppression therapy and surgical procedure had been established, the field of PLT hit a bottleneck. PLT is facing increasing long-term problems such as hepatitis-related fibrosis (3), which are leading to the loss of quality of life. However, in CiteSpace, we found that few studies made great progress in solving these problems. According to Thomas Kuhn's Structure of Scientific Revolutions, this field may be between a normal science and a science in crisis (43). Studies that can resolve the crisis, or in other words, answer the top concerns in the field of PLT, will be the new intellectual milestones. New intellectual milestones may occur as innovations in immunosuppression therapy or liver cell transplantation.

Some limitations were observed in this research. First, almost all of the bibliometric analyses may be limited by the source of the retrieval. In our study, that is, the Web of Science. Although the Web of Science Core Collection contains the world's leading scholarly journals, books, and proceedings in every field, it does not include enough non-English databases. Besides, the search strategy used is in English, which means many non-English researches are excluded. For example, nowadays, many Asian countries have also achieved great progress in PLT, such as Japan (22), South Korea (44), and China (26). Therefore, non-inclusion of research from these countries may induce bias. Secondly, it must be pointed out that although bibliometric analysis allows viewing the PLT field from different perspectives and presents some quantitative results, it gives limited information on each research theme. To obtain more information, we should not skip data searching and reading of the articles. For example, when we tried presenting the intellectual milestones, we just gave a title table and summarized the main points of these articles, which contained few details.

## Conclusions

We presented a bibliometric review of the articles concerning PLT and analyzed the publication trends, hotspots, research fronts, and intellectual milestones. In terms of publication trends, studies on PLT steadily increased. PITT is the most prolific institution in the field of PLT. The three most productive research regions are North America, Europe, and East Asia. Currently, interdisciplinary studies on PLT are scarce. In terms of hotspots and research fronts, over the past two decades, researchers' main efforts have transferred from improving survival rates to improving long-term outcomes. Thus, studies of observation of long-term outcomes, expanding the indications, risk factors of the outcome of PLT, and cell-based therapies, can be new research fronts. Shortage of intellectual milestones may be the result of a lack of new therapies. Researches that can resolve this problem will have the potential to be new intellectual milestones.

Based on the above results, we suggest that: (1) developing countries have accumulated a large number of PLT cases in recent years, which can provide a large sample size. We suggest that researchers from developed and developing countries should cooperate more to better promote the research progress of PLT. (2) considering the lack of interdisciplinary studies, conducting interdisciplinary studies on PLT may be promising, since it will help solve the problems of biomaterials, model constructions, organ allocation, etc. in PLT. (3) clinical studies on the outcome of PLT should be more inclined to choose long-term outcome as the end point. (4) future basic researchers should focus more on exploring liver cell transplantation, new immunosuppressant and immunosuppression withdrawal.

## Author Contributions

LS and JZ designed and conducted the study, analyzed the data, drafted the initial manuscript, and revised the manuscript. WD and CJ designed and conducted the study, analyzed the data, and reviewed the manuscript. DS conceptualized and designed the study, coordinated and supervised data collection, and critically reviewed the manuscript for important intellectual content. WY and QX designed the study, coordinated and supervised data collection, and critically reviewed the manuscript for important intellectual content.

## Funding

This study was supported by grants from the National Natural Science Foundation of China (Nos. 81771133 and 81970995), Shanghai Shenkang Hospital Development Center Founding (SHDC12017X11), Shanghai Municipal Science and Technology Commission Founding (21S31900100), Renji Hospital Clinical Innovation Foundation (PYII20-09), and Shanghai municipal Education Commission-Gaofeng Clinical Medicine Support (20191903). The funders had no role in the analyses and interpretation of the results or writing of the manuscript.

## Conflict of Interest

The authors declare that the research was conducted in the absence of any commercial or financial relationships that could be construed as a potential conflict of interest.

## Publisher's Note

All claims expressed in this article are solely those of the authors and do not necessarily represent those of their affiliated organizations, or those of the publisher, the editors and the reviewers. Any product that may be evaluated in this article, or claim that may be made by its manufacturer, is not guaranteed or endorsed by the publisher.

## Abbreviations

CsA: cyclosporine A
FK 506: tacrolimus
LDLT: living donor liver transplantation
MLDs: metabolic liver diseases
PITT: University of Pittsburgh
PLT: pediatric liver transplantation.
